# Dose response kinetics of CD8 lymphocytes from young animals transfused into old animals and challenged with influenza

**DOI:** 10.1186/1742-4933-10-34

**Published:** 2013-08-16

**Authors:** Richard Aspinall, Sheila Govind, Antonio Lapenna, Pierre Olivier Lang

**Affiliations:** 1 Regenerative Medicine Group, Cranfield Health, Cranfield, UK; 2 Nescens Centre of preventive medicine, Clinique of Genolier, Genolier, Switzerland; 3 Translational Medicine, Cranfield Health, Cranfield University, Cranfield MK430AL, UK

**Keywords:** Influenza virus, CD8 T cell, Transfusion

## Abstract

Transfusion of autologous leukocytes after prolonged storage has been proposed as a means of rejuvenating the immune system of older individuals. The rationale for this approach is that age related immune decline is associated with a diminished pool of naïve T cells following atrophy of the thymus and reduction in thymic output. The presence of high levels of naïve T cells within the blood of young individuals could provide a boost to the immune system of an older “self” through a rejuvenation of the naïve T cell pool. However what remains unresolved is whether the cells could be incorporated effectively into the T cell pool of the host and whether effectors could be generated. Using CD45 congenic mice in our experiments we show that the transfusion of young donor cells into older congenic host animals leads to their successful incorporation into the peripheral T cell pool. When the recipients were challenged with influenza virus, specific effector CD8 cells were generated which were of both host and donor origin. We found no relationship between the number of responder cells of donor origin at the time of assay and the number of cells injected.

## Background

Influenza is an infectious disease of limited duration in young adults with an optimal functioning immune system. It is normally associated with a sudden onset and symptoms which can include fever, cough, nasal congestion, and aching joints. In older individuals, whose immune system shows a degree of age-related dysfunction, influenza infection has serious consequences including an increased risk of mortality and a greater likelihood of hospitalization, but also acts as a trigger for functional decline [1-3].

Evidence for this decline, often termed immunosenescence, has been derived from data gained from epidemiological studies, results from vaccine trials and laboratory investigations, and observations by clinicians. Epidemiological studies reveal that the prevalence of diseases affecting epithelial barriers, such as respiratory and urinary tract infections, considerably increases with age. Moreover the ability to control persistent viral infection also diminishes as we age [2,4,5]. Murine models have shown that age associated immune decline has multiple causes, which include a much smaller pool of naïve T cells following reduced output from the thymus as a consequence of age associated thymic atrophy, diminished functional performance of the peripheral T lymphocytes, specifically the CD8 cells, and the presence of holes in the repertoire arising from the loss of some clonotypes [6-10]. These changes may be central to the issue of making an effective response to influenza viruses, since previous studies have shown the critical role of CD8 cells in the control of viral infections. Early studies identified that cloned CD8 cells specific for influenza viruses, when transferred into influenza infected mice, could reduce both lung virus titers, prolong survival times, [11] and in addition could protect mice from lethal infections [12].

Improvements in the CD8 T cell pool of old animals, can be achieved by increasing the number of reactive cells through adoptive transfer. This was tested in a recent study using virus-specific transgenic CD8 T cells taken from young animals, and transfused into old animals. The results revealed that the environment in the old animals inhibited their clonal expansion during virus infection [13]. A different approach was taken in experiments to increase specific influenza virus T cell responses, through providing a larger naïve T cell pool in old animals by reversing thymic atrophy using interleukin 7. This rejuvenation of the immune system has produced old animals that show improved responses to influenza viral infection with much reduced viral loads in the lungs, compared with age matched controls [14]. One of the issues with treatment with interleukin 7 is the need for frequent injections [15-17]. Thus, identifying new alternative approaches to rejuvenate the immune system, specifically the naïve T cell pool, is an attractive challenge. One option which has been proposed is to increase the number of naive T lymphocytes in the peripheral T cell pool by transfusion [18]. This could be achieved by the preservation of quantities of an individual’s blood leukocytes at a younger age, which would then be transfused back into them at a later stage, when the immune system demonstrates some features of immunosenescence. Such use of autologous tissue would, of course, negate any problems associated with rejection. Adoptive transfer of lymphocytes has already been suggested for the treatment of some viral diseases [19] and cancers [20]. The transfer of syngeneic or autochthonous T cells from young to old whilst having been suggested [18] has not been tested.

Two major overlapping issues confront the idea of rejuvenating an older individual’s immune system by transfusing blood leukocytes stored from when they were younger. The first is, whether an individual whose immune system is compromised, would be able to provide a suitable environment for any transfused cells to make an immune response. The second issue is how many leukocytes have to be infused in order to ensure that sufficient specific effectors are generated.

In order to clarify these two major issues, we transferred blood from young C57BL/6.SJL mice into old C57BL/6 mice and then vaccinated them against influenza infection (three injections of formalin inactivated influenza virus A/PR/8 over two weeks) before challenging them 3 weeks after the last vaccination with live A/PR/8 influenza virus delivered intra-nasally. We transferred graded amounts of blood into the old animals, and assessed the quantity of virally specific CD8 T cells by phenotypic staining.

Peripheral blood constitutes approximately 1-2% of the total T cell pool and samples taken from blood are often considered to be representative of the total T cell pool. To parallel potential future manipulations in the rejuvenation of the human immune system we have chosen to derive our donor cells from the blood of young mice.

## Results

### Young blood leukocyte constituents

**Figure 1:**
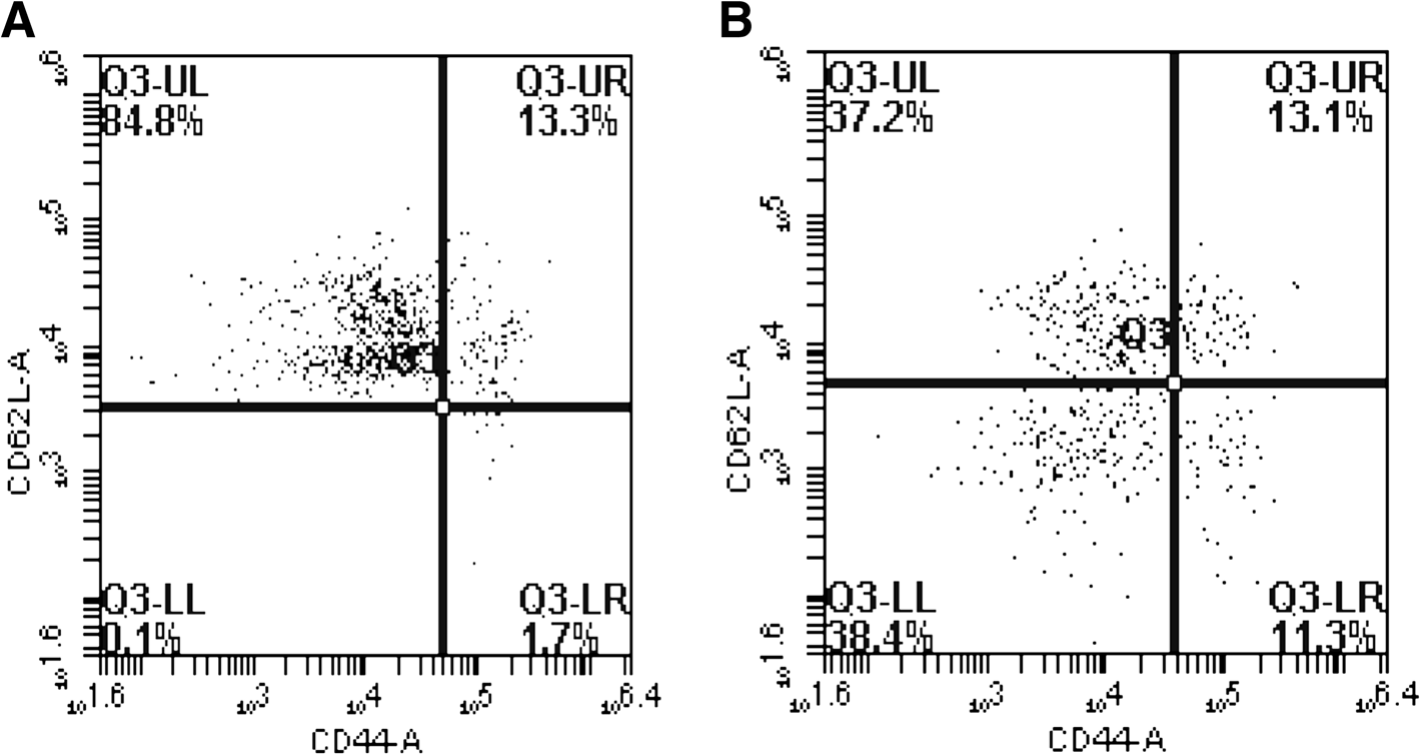
**Flow cytometer dot plot of the naive (CD62L**^**hi **^**CD44**^**lo**^**) and memory (CD62L**^**lo **^**CD44**^**hi**^**) cells which were CD3**^**+**^**CD4**^**+**^** (A) or CD3**^**+**^**CD8**^**+**^** (B) in the blood of young donor animals.**

**Figure 2:**
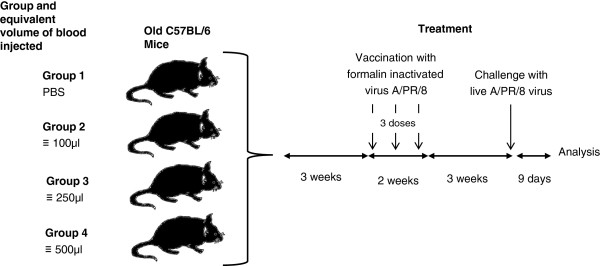
A schematic of the experimental design.

Analysis of the blood collected from young animals revealed that the mononuclear leukocyte population contained 65% CD3^+^ cells of which 33% were CD8^+^. Within the latter population 58% were naive cells with a phenotype of CD3^+^CD8^+^CD44^-^CD62L^+^ whilst 16% had a memory phenotype of CD3^+^CD8^+^CD44^+^CD62L^-^. Figure 1 shows flow cytometer dot plots of phenotypes within the CD4^+^ and CD8^+^ subsets. Cell counts on the blood revealed that there was on average 7.1 × 10^6^ cells per ml. Mononuclear cells were prepared from the blood and transfused into recipient older animals. Four groups of older animals were used: group 1 received no cells but saline vehicle alone; group 2 received 7.1 × 10^5^ cells equivalent to 100 μl of blood; group 3 received 1.8 × 10^6^ cells equivalent to 250 μl of blood; and groups 4 received 3.5 × 10^6^ cells equivalent to 500 μl of blood. Table 1 shows the numbers and phenotypes of populations of cells received by each group. These cells were transferred into the old host and a schematic of the procedure used is shown in Figure 2.

**Table 1:** Numbers and phenotypes of cells received by each group of recipient old animals

**Phenotype of cells**	**Group 1 animals**	**Group 2 animals**	**Group 3 animals**
Total number of cells injected	710,000	1,800,000	3,500,000
Number ofCD3^+^ cells injected	461,500	1,170,000	2,275,000
Number of CD3^+^CD8^+^ cells injected	152,295	386,100	750,750
Number of CD3^+^CD8^+^CD62L^+^CD44^−^ cells injected	88,331	223,938	435,435
Number of CD3 + CD8 + CD62L^−^CD44^+^ cells injected	24,367	61,776	120,120

### Effect on cell numbers in recipients

Our analysis of the four groups of old mice revealed that we had successfully introduced young donor T cells into these animals. We identified the origin of the cells using anti-CD45.1 staining. Cells of host origin, from the old mice, would appear to be CD45.1^-^ whilst those of donor origin from the young mice would be CD45.1^+^. In all figures the cells of host origin are represented by filled circles and those of donor origin by open circles.

**Figure 3:**
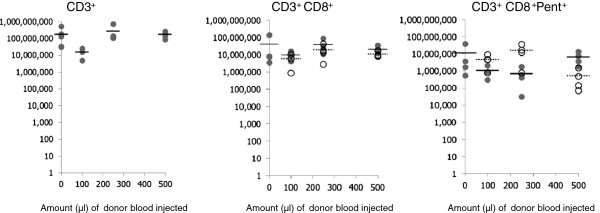
**Numbers of T cell subsets in the spleens of individual mice within each of the experimental groups.** Open circles (donor origin) are CD45.1^+^ and filled circles (host origin) CD45.1^-^ cells. Solid bars indicate average numbers of cells of host origin and dashed bars indicate average numbers of cells of donor origin.

**Figure 4:**
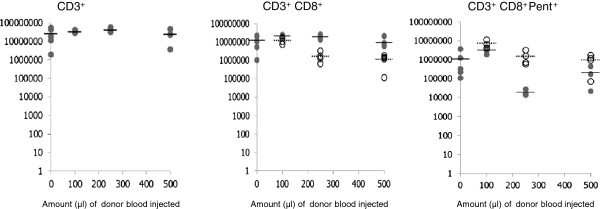
**Numbers of T cell subsets in the lymph nodes of individual mice within each of the experimental groups.** Open circles (donor origin) are CD45.1^+^ and filled circles (host origin) represent CD45.1^-^ cells. Solid bars indicate average numbers of cells of host origin and dashed bars indicate average numbers of cells of donor origin.

**Figure 5:**
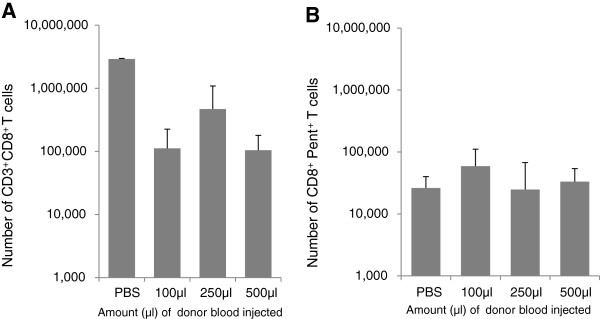
**Numbers of CD3**^**+**^**CD8**^**+**^** T cells (A) and CD8**^**+**^**Pentamer**^**+**^** T cells (B) in the left lung of mice within each of the experimental groups.** No significant difference (p > 0.05) was found between PBS values and any of the treatment groups in either **(A)** or **(B)**.

Figures 3 and 4 show the numbers of CD3^+^ and CD3^+^CD8^+^ cells in the spleens and lymph nodes of the old recipients at the time of assay. The results reveal that irrespective of the number of cells injected, the overall numbers of CD3^+^ T cells in these organs remained within the same boundaries and were not significantly different from control animals who received no cells. Similar results were seen for the CD3^+^CD8^+^ T cells. Analysis of the numbers of donor derived CD3^+^CD8^+^ cells in the host revealed that despite there being considerable differences in the number of cells injected into the host, there was no significant difference in their final numbers in either the lymph node or the spleens (Figures 3 and 4). Within the spleen the percentage of donor CD8^+^ cells (CD45.1^+^) of the total CD8^+^ population was extremely similar at 32, 31, and 37% for the recipients of 100, 250 and 500 μl equivalent of blood respectively. Analysis of the lungs revealed that the number of CD3^+^CD8^+^ T cells was greater in those animals receiving no transfused cells but similar in those to whom cells were transfused (Figure 5A).

### Cellular immune responses

Analysis of the number of virus specific CD3^+^CD8^+^Pentamer^+^ T cells in the spleen and lymph nodes revealed that in each treatment group the numbers of these cells in the spleens and lymph nodes were similar (Figures 3 and 4). Moreover where transfusion of cells had occurred there were similar numbers of virus specific cells of donor and host origin in these organs. Analysis of the lungs revealed that the number of CD8^+^Pentamer^+^ cells in the lungs were similar (Figure 5B).

### CD107a

**Figure 6:**
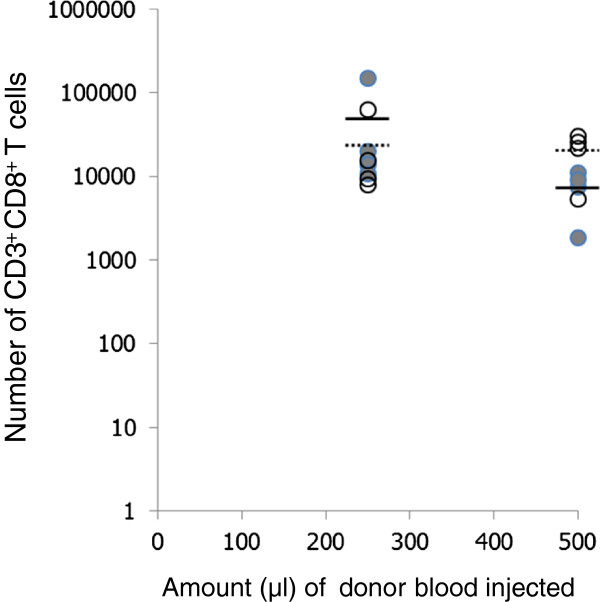
**Numbers of CD3**^**+**^**CD8**^**+**^**CD107**^**+**^**Pentamer**^**+**^** cells in lungs of individual animals in treatment groups receiving either 250 or 500 μl of blood.** Open circles (donor origin) are CD45.1^+^ and filled circles (host origin) represent CD45.1^-^ cells. Solid bars indicate average numbers of cells of host origin and dashed bars indicate average numbers of cells of donor origin.

Target cells are lysed by effector CD8^+^ cells through a process involving release of granules containing granzyme and perforin into the synapse between effector and target cell. Following this process glycoproteins which were once found on the lysosomal membrane are expressed on the surface. One of these proteins is CD107a, therefore a cell expressing CD107a must have undergone degranulation following target interaction [21]. Analysis of the cells in the lungs of animals which received either 250 μl or 500 μl blood equivalent are shown in Figures 4 and 5. The numbers of cells in the lungs were similar. When we analysed these cells further, in animals which had received the equivalent of 250 or 500 μl of blood, in order to determine whether they were of host or donor origin, the results (Figure 6) showed similar numbers of each of these in animals.

### Viral load

**Figure 7:**
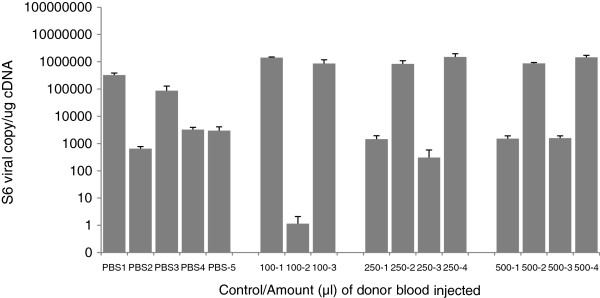
**Absolute qPCR quantification of viral load in lung tissue.** Viral load detection was performed by qPCR using a standard curve to obtain the number of copies of viral RNA per μg of cDNA sample. Columns represent average viral copies obtained from triplicate analysis.

We measured absolute viral load in lung tissue using real time quantitative PCR (Figure 7), and found that whilst the viral load amongst the animals receiving PBS vehicle alone was relatively similar with an average of 3.2 × 10^4^. In contrast the viral load for the treated animals appears variable. In the recipients of higher doses half of the animals have viral loads which are less than 2000 and the other half of the readings are closer to 10^6^. Although we cannot completely discount technical reasons for such variability, it may also indicate the individual variability amongst animals either in the cells they received or the environment provided and whether this was able to support an effective response.

## Discussion

This study reveals that cells from young animals transferred to an older congenic host can be incorporated into the peripheral T lymphocyte pool without having a significant effect on the total number of CD3^+^ T cells. In addition, the donor cells within the host are able to undergo antigen-induced proliferation and differentiation to produce effector CD8 cells, which could contribute to the response to influenza infection *in vivo*, in a manner similar to that seen with the host cells. This would suggest that the environment provided by the host is not lacking and that effectors could be generated in an immune response to antigenic challenge. However the functional response as judged by viral load would appear to be variable, muted in some animals and showing greater effectiveness in others.

The total blood volume of an average mouse is 2 millilitres whilst the average human contains approximately 5 litres [22]. In our experiments we have transferred the equivalent of 100 μl, 250 μl or 500 μl of blood from young into old mice, which is the equivalent of transferring 250, 625 or 1250 ml of blood from a young human into an older host. Adoptive cell transfer has been used previously as a form of immunotherapy for patients with certain cancers or chronic infections. The therapy normally involves the expansion of specific T cells *ex vivo* and their transfer into hosts, which have been treated to deplete resident T lymphocytes in order to create “space” for the transferred cells. The treatment to induce lymphopenia is often associated with unwanted toxicity. In these experiments we have transferred blood derived leukocytes into lymphoreplete hosts without prior selection or expansion. Entry and survival of the introduced cells in the resident pool demands their competition with resident cells for specific niches and necessary cytokines and is not driven by exogenous antigen [23,24]. Our adoptively transferred cells were given 3 weeks before meeting influenza antigen as previous work indicated that this is the time necessary to enter the peripheral pool [25,26]. After the introduction of antigen both resident and transferred cells would be driven to proliferate, competing for available resources. Our results reveal that although there was a five-fold difference between the lowest and the highest number of cells transferred, at the time of assay there was no major difference in the numbers of donor cells in the hosts when compared with the numbers injected. Our experiments would suggest that there appears to be no direct correlation between the number of cells injected and the number of cells present within the host at the time of assay, implying that cell dose was not a critical factor in incorporation into the peripheral T cell pool.

Failure to correlate initial cell dose with final cell numbers suggests considerable expansion from the injected cells at the lower doses compared with the higher dose. Our results suggest that in the spleen there was on average a 38-fold expansion of the CD3^+^CD8^+^ injected cells in those animals receiving the equivalent of 100 μl of blood and for those receiving the 250 μl equivalent there was on average a 50-fold expansion. This is before taking into account the cells in the lymph nodes and the rest of the immune system. However this is not accompanied by a dramatic increase in the overall number of cells indicating that there must be niche competition between cells of donor and host origin.

The concept that inadequate responses to vaccines are made in the elderly because of a lack of diversity within the peripheral T cell repertoire has been common currency [10]. The size of the αβ T cell repertoire in the spleens of young mice has been calculated to be in the region of 2 × 10^6^ clones with approximately 10 cells per clone [27]. Whilst rejuvenation of the repertoire in older individuals through reversal of thymic atrophy and increasing thymic output leads to improved responses and a possible broader repertoire, providing an improved/younger repertoire through the transfusion of lymphocytes may not “fill” the holes in the repertoire. Our results in this study using a dose escalation of lymphocytes would suggest that the functional repertoire within the lowest dose was in the region of 15,200 clones. Either this number of clones contains the receptor for the A/PR/8 NP 366–374 (ASNENMETM) peptide or else there is considerable cross reactivity within the repertoire which has been suggested previously [28].

Failure to see significant decline in viral load in the lungs of all treated animals in the presence of an apparently active CD8 mediated response was unexpected. This may be related to a host versus graft immune response, which is not without precedent having been reported previously [29]. Indeed some recipient animals appeared to show structures which resembled abnormally large lymph nodes and our analysis of these revealed they contained cells of host origin the majority of which were of a memory phenotype (CD62L^-^CD44^+^) and many of them appear to be CD4^+^ (data not shown). The degree of viral clearance whilst not correlating with CD8 effector cell numbers in the lung may be related to an on-going host versus graft response in the animals.

## Conclusions

Transfusion of young donor cells into older congenic host animals leads to their incorporation into the peripheral T cell pool. The generation of effector cells from the cell population injected would suggest that they are contributing to the overall immune response in the animal. However in this pilot study the young donor cells appear to have no clear overall additional effect on the hosts response to challenge with influenza virus.

## Materials and methods

### Mice

C57BL/6 (CD45.2) female mice were maintained by Charles Rivers Laboratories until they were 22 months of age. C57BL/6.SJL (CD45.1) female mice were obtained from Charles Rivers when 6 weeks of age. Both were sent to the Biological Research Unit where these experiments were undertaken. All experiments were carried out in accordance with the local rules and regulations and under a project license reviewed by the Open University review board and approval by the Home Office.

### Treatment

#### Young mice

The C57BL/6.SJL (CD45.1) female mice were exsanguinated at 6 weeks of age and the blood collected into tubes containing EDTA as an anticoagulant. Mononuclear cells were isolated by density dependant centrifugation, washed by centrifugation and then counted. These cells were frozen in freezing mixture consisting of FCS supplemented with 10% DMSO and stored at −150°C until required.

#### Old mice

The 22 month old C57Bl/6 female mice were injected in the tail vein with cells from young (6 week old) female C57BL6/SJL female mice according to the following regimes:

Group 1 received the equivalent of 100 μl of blood

Group 2 received the equivalent of 250 μl of blood

Group 3 received the equivalent of 500 μl of blood

Group 4 received saline vehicle alone

The mice were left for 3 weeks and then vaccinated with formalin inactivated influenza virus (A/PR/8) receiving three injections over a 2 week period and then 3 weeks after the last vaccination the mice were challenged with approximately 50 pfu of live A/PR/8 influenza virus delivered intra-nasally. They were monitored over the next eight days and culled on the ninth day and their tissues removed and analysed. Mice were therefore over 24 months of age at the time of analysis. Initially each group of mice contained 5 animals but of the 20 animals which started the experiment only 16 were analysed as the remaining 4 died at different stages during the procedure.

### Cell preparation from lymph nodes, spleen and lung

Axial and mesenteric lymph nodes and spleen were excised and the organs placed in media consisting of DMEM and PBS supplemented with 5% FCS. The tissues were taken to the laboratory at Cranfield where cell suspensions were made by passing the tissue through a cell strainer (Becton Dickinson, Oxford, U.K.) into DMEM plus PBS supplemented with 5% FCS. In the spleen cell suspensions the erythrocytes were lysed using red cell lysis fluid (Ortho, Amersham, U.K.), and total number of cells then counted. The cells were washed by centrifugation and resuspended in freezing media consisting of FCS with 10% DMSO and frozen and stored at −150°C until analysed.

For the lung tissues, both lungs were removed and the cells suspensions made from the left lobe by passing the lobe through a cell strainer (Becton Dickinson, Oxford, U.K.) into DMEM plus PBS supplemented with 5% FCS. The cells were counted and then washed by centrifugation and frozen in the freezing mixture.

### Staining lymph node and spleen cells

These cells were defrosted and washed in DMEM and PBS supplemented with 5% FCS and approximately 10^6^ cells in were stained with the following antibody cocktail anti CD45.1 PE-Cy5 (clone A20), anti-CD8 APC (clone 53–6.7), anti CD3 FITC (clone 145 2C11) (all from Cambridge BioSciences) and PE conjugated pentamer specific for anti H-2Db Influenza A/PR/8 NP 366–374 (ASNENMETM) peptide (ProImmune). In addition a further 10^6^ cells were stained with the isotype control for each antibody or the pentamer control. For staining the cells were incubated in a volume of 100 μl for approximately 45 minutes at 4°C in the wells of a round bottomed 96 well plate before being washed by centrifugation and resuspended in 1% solution of paraformaldehyde in PBS. Analysis was carried out on an Accuri C6 flow cytometer.

### Staining lung cells

These cells were defrosted and washed in DMEM and PBS supplemented with 5% FCS and approximately 10^6^ cells in were stained with the following antibody cocktail anti CD45.1 PE-Cy5 (clone A20), anti-CD8 APC (clone 53–6.7), anti CD3 FITC (clone 145 2C11) and PE conjugated pentamer specific for anti H-2Db Influenza A/PR/8 NP 366–374 (ASNENMETM) peptide. A separate aliquot of 10^6^ cells was stained with the following antibody cocktail anti CD45.1 PE-Cy5 (clone A20), anti-CD8 APC (clone 53–6.7), anti CD107a FITC (clone H4A3) and PE conjugated pentamer specific for anti H-2Db Influenza A/PR/8 NP 366–374 (ASNENMETM) peptide. In addition two further lots of 10^6^ cells were stained with the isotype control for each antibody or the pentamer control where appropriate. Staining was carried out as described above. Analysis was carried out on an Accuri C6 flow cytometer.

### Staining blood leukocytes from young animals

The leukocytes from the C57BL/6.SJL young animal were stained with the following antibody cocktail anti-CD44 APC (clone IM7) anti-CD62L PerCP (clone Mel 14) anti CD3 FITC (clone 145 2C11) and either anti-CD4 PE or anti-CD8 PE. The staining process was as described above. Analysis was carried out on an Accuri C6 flow cytometer.

### Viral load

To determine viral load, 20-30 mg of frozen lung tissue was suspended in buffer RTL (Qiagen), disrupted for 20 sec at 6500 rpm with CK28 ceramic beads using the Percellys 24 tissue homogenizer (Stretton Scientific). RNA extraction was performed on the supernatant according to the manufacturer’s guidelines including treatment with DNAse1 prior to elution (RNeasy Qiagen). RNA integrity was quantified using the BioRad Experion. Samples with RQI values of 7.5 and above were used for further analysis. RNA concentrations were determined using the Picodrop and normalised to 1000 ng/10 μl for subsequent MultiScribe™ reverse transcriptase reaction to generate cDNA using the High Capacity Reverse transcriptase kit (Applied Biosystems). Absolute quantification of viral copy number was performed using a log linear 10^1^-10^7^ standard curve using a viral RNA plasmid construct of region 855-999 bp of Segment 6 neuraminidase (NA) gene [30]. qPCR reactions were set up in triplicate using the QIAgility and performed on the Rotorgene Q (Qiagen). Standard cycling conditions were hot start at 95°C for 15 mins, and 40 cycles of 95°C for 15 sec, 55°C for 30 sec, and 72°C for 30 sec. The 10 μl reaction mix comprised of 5 μl of 2×Quantitect SYBR (Qiagen), 0.5 μM forward (855–874) ATTTGCCTATGAGACCGATGCT and reverse (975–999) AGGATGGGGGCTGTGACC [30], 2 μl of cDNA equivalent to 100 ng and 2 μl of water. The specificity of the qPCR product was validated by melt curve analysis.

## Competing interests

RA is a consultant for Future Health.

## Authors’ contributions

RA designed the experiments, undertook some of the animal work, stained the cells and wrote the manuscript with POL and SG. Both RA and POL carried out the statistical analysis of the data. SG carried out the viral load analysis and AL undertook the fluorescent cell analysis. All authors read and approved the final manuscript.

## References

[B1] Lang PO Aspinall R Immunosenescence and herd immunity: with an ever-increasing aging population do we need to rethink vaccine schedules? Expet Rev Vaccine 2012 11 2 167 176 10.1586/erv.11.187 22309666

[B2] Lang PO Govind S Michel JP Aspinall R Mitchell WA Immunosenescence: implications for vaccination programmes in adults Maturitas 2011 68 4 322 330 10.1016/j.maturitas.2011.01.011 21316879

[B3] McElhaney JE Influenza vaccination in the elderly: seeking new correlates of protection and improved vaccines Aging health 2008 4 6 603 613 10.2217/1745509X.4.6.603 20011611 PMC2790215

[B4] Monto AS Ansaldi F Aspinall R McElhaney JE Montano LF Nichol KL Puig-Barbera J Schmitt J Stephenson I Influenza control in the 21st century: optimizing protection of older adults Vaccine 2009 27 37 5043 5053 10.1016/j.vaccine.2009.06.032 19559118

[B5] McElhaney JE Zhou X Talbot HK Soethout E Bleackley RC Granville DJ Pawelec G The unmet need in the elderly: how immunosenescence, CMV infection, co-morbidities and frailty are a challenge for the development of more effective influenza vaccines Vaccine 2012 30 12 2060 2067 10.1016/j.vaccine.2012.01.015 22289511 PMC3345132

[B6] Pido-Lopez J Imami N Andrew D Aspinall R Molecular quantitation of thymic output in mice and the effect of IL-7 Eur J Immunol 2002 32 10 2827 2836 10.1002/1521-4141(2002010)32:10<2827::AID-IMMU2827>3.0.CO;2-X 12355435

[B7] Decman V Laidlaw BJ Doering TA Leng J Ertl HC Goldstein DR Wherry EJ Defective CD8 T cell responses in aged mice are due to quantitative and qualitative changes in virus-specific precursors J Immunol 2012 188 4 1933 1941 10.4049/jimmunol.1101098 22246631 PMC3320034

[B8] Decman V Laidlaw BJ Dimenna LJ Abdulla S Mozdzanowska K Erikson J Ertl HC Wherry EJ Cell-intrinsic defects in the proliferative response of antiviral memory CD8 T cells in aged mice upon secondary infection J Immunol 2010 184 9 5151 5159 10.4049/jimmunol.0902063 20368274

[B9] Ahmed M Lanzer KG Yager EJ Adams PS Johnson LL Blackman MA Clonal expansions and loss of receptor diversity in the naive CD8 T cell repertoire of aged mice J Immunol 2009 182 2 784 792 19124721 10.4049/jimmunol.182.2.784 PMC2724652

[B10] Yager EJ Ahmed M Lanzer K Randall TD Woodland DL Blackman MA Age-associated decline in T cell repertoire diversity leads to holes in the repertoire and impaired immunity to influenza virus J Exp Med 2008 205 3 711 723 10.1084/jem.20071140 18332179 PMC2275391

[B11] Lin YL Askonas BA Biological properties of an influenza A virus-specific killer T cell clone. Inhibition of virus replication in vivo and induction of delayed-type hypersensitivity reactions J Exp Med 1981 154 2 225 234 10.1084/jem.154.2.225 6267157 PMC2186413

[B12] Lukacher AE Braciale VL Braciale TJ In vivo effector function of influenza virus-specific cytotoxic T lymphocyte clones is highly specific J Exp Med 1984 160 3 814 826 10.1084/jem.160.3.814 6206190 PMC2187390

[B13] Jiang J Bennett AJ Fisher E Williams-Bey Y Shen H Murasko DM Limited expansion of virus-specific CD8 T cells in the aged environment Mech Ageing Dev 2009 130 11–12 713 721 19744506 10.1016/j.mad.2009.08.007 PMC2839881

[B14] Henson SM Snelgrove R Hussell T Wells DJ Aspinall R An IL-7 fusion protein that shows increased thymopoietic ability J Immunol 2005 175 6 4112 4118 16148161 10.4049/jimmunol.175.6.4112

[B15] Rosenberg SA Sportes C Ahmadzadeh M Fry TJ Ngo LT Schwarz SL Stetler-Stevenson M Morton KE Mavroukakis SA Morre M IL-7 administration to humans leads to expansion of CD8+ and CD4+ cells but a relative decrease of CD4+ T-regulatory cells J Immunother 2006 29 3 313 319 10.1097/01.cji.0000210386.55951.c2 16699374 PMC1473976

[B16] Sportes C Babb RR Krumlauf MC Hakim FT Steinberg SM Chow CK Brown MR Fleisher TA Noel P Maric I Phase I study of recombinant human interleukin-7 administration in subjects with refractory malignancy Clinical cancer research: an official journal of the American Association for Cancer Research 2010 16 2 727 735 10.1158/1078-0432.CCR-09-1303 20068111 PMC2808195

[B17] Sportes C Hakim FT Memon SA Zhang H Chua KS Brown MR Fleisher TA Krumlauf MC Babb RR Chow CK Administration of rhIL-7 in humans increases in vivo TCR repertoire diversity by preferential expansion of naive T cell subsets J Exp Med 2008 205 7 1701 1714 10.1084/jem.20071681 18573906 PMC2442646

[B18] Charron D Autologous white blood cell transfusion: toward a younger immunity Hum Immunol 2007 68 10 805 812 10.1016/j.humimm.2007.07.004 17961768

[B19] Fujita Y Rooney CM Heslop HE Adoptive cellular immunotherapy for viral diseases Bone Marrow Transplant 2008 41 2 193 198 10.1038/sj.bmt.1705906 17982497

[B20] June CH Principles of adoptive T cell cancer therapy J Clin Investig 2007 117 5 1204 1212 10.1172/JCI31446 17476350 PMC1857246

[B21] Aktas E Kucuksezer UC Bilgic S Erten G Deniz G Relationship between CD107a expression and cytotoxic activity Cell immunol 2009 254 2 149 154 10.1016/j.cellimm.2008.08.007 18835598

[B22] Mestas J Hughes CC Of mice and not men: differences between mouse and human immunology J immunol 2004 172 5 2731 2738 14978070 10.4049/jimmunol.172.5.2731

[B23] Paulos CM Suhoski MM Plesa G Jiang T Basu S Golovina TN Jiang S Aqui NA Powell DJ Jr Levine BL Adoptive immunotherapy: good habits instilled at youth have long-term benefits Immunol Res 2008 42 1–3 182 196 18949448 10.1007/s12026-008-8070-9 PMC3809041

[B24] Tchao NK Turka LA Lymphodepletion and homeostatic proliferation: implications for transplantation American journal of transplantation: official journal of the American Society of Transplantation and the American Society of Transplant Surgeons 2012 12 5 1079 1090 10.1111/j.1600-6143.2012.04008.x 22420320

[B25] Berzins SP Godfrey DI Miller JF Boyd RL A central role for thymic emigrants in peripheral T cell homeostasis Proc Natl Acad Sci USA 1999 96 17 9787 9791 10.1073/pnas.96.17.9787 10449772 PMC22288

[B26] Berzins SP Boyd RL Miller JF The role of the thymus and recent thymic migrants in the maintenance of the adult peripheral lymphocyte pool J Exp Med 1998 187 11 1839 1848 10.1084/jem.187.11.1839 9607924 PMC2212318

[B27] Casrouge A Beaudoing E Dalle S Pannetier C Kanellopoulos J Kourilsky P Size estimate of the alpha beta TCR repertoire of naive mouse splenocytes J Immunol 2000 164 11 5782 5787 10820256 10.4049/jimmunol.164.11.5782

[B28] Mason D A very high level of crossreactivity is an essential feature of the T-cell receptor Immunol Today 1998 19 9 395 404 10.1016/S0167-5699(98)01299-7 9745202

[B29] Xu H Exner BG Chilton PM Schanie C Ildstad ST CD45 congenic bone marrow transplantation: evidence for T cell-mediated immunity Stem cells 2004 22 6 1039 1048 10.1634/stemcells.22-6-1039 15536194

[B30] Vester D Lagoda A Hoffmann D Seitz C Heldt S Bettenbrock K Genzel Y Reichl U Real-time RT-qPCR assay for the analysis of human influenza A virus transcription and replication dynamics J Virol Meth 2010 168 1–2 63 71 10.1016/j.jviromet.2010.04.017 20433869

